# Thoughts for Young Men

**Published:** 1870-05

**Authors:** 


### THOUGHTS FOR YOUNG MEN.

"As I walked by myself, I talked to myself,
Myself he said unto me:
Beware of thyself, take care of thyself,
For nobody cares for thee."—OLD SONG.

And "Myself," though often a little contracted in his views, made a sensible speech that time, and showed good judgment in selecting an appreciative audience; for he found "me" an attentive listener, who believed every word he said. The advice is not needed by a majority of our race or profession. They are generally sufficiently selfish. This may be thought uncharitable, especially when applied to what we so complacently term "our glorious profession." But it is well to face the facts. Our acquaintance with the Dental profession is somewhat extended, and we don't know twenty Dentists who would really care if the best man in the profession should die, only so far as they hope and expect to use him. And our profession is not worse than other people. "Nobody cares when a bachelor dies," and very few care when anybody else dies. And why should they? It makes but little difference *when* a man dies. *How* he dies is much more important—second only to *how he lives*.

A due appreciation of these principles will not make you selfish, reserved, and negligent in the pursuit of professional knowledge. Science rewards its votaries directly. You should cultivate it for its own sake, and that you may the better fulfill the end of your creation, in view of the great principal that "no man liveth unto himself." Science cultivated with this view will bless the possessor and benefit the race. Cultivated with the expectation of compensatien, or even gratitude from the profession, will yield only a harvest of dead sea apples.

A few days ago, by the aid of an almanac and a family record, we found ourselves old. It is a duty of age to counsel youth. We hasten to comply, and freely give this, which is as sincere as it is gracious. "Blessed are they that expect nothing, for they can stand it to be disappointed." W.

